# Translational approaches to coagulopathy after trauma: Towards targeted treatment

**DOI:** 10.1371/journal.pmed.1002359

**Published:** 2017-07-25

**Authors:** Mitchell Jay Cohen

**Affiliations:** 1 Denver Health Medical Center, Denver, Colorado, United States of America; 2 University of Colorado School of Medicine, Aurora, Colorado, United States of America

## Abstract

Mitchell J. Cohen discusses why trauma care must go beyond restoring perfusion to target disorders of inflammation and coagulation in severely injured patients.

Understanding of the physiology of hemostasis after injury and shock has evolved in recent years as a result of 2 parallel and synergistic lines of investigation. First, the recognition that acute traumatic coagulopathy (ATC, also called trauma-induced coagulopathy [TIC]) represents an endogenous perturbation of coagulation set in motion a number of investigations into the fundamental driving biology and physiology behind ATC [1–3]. Second, the concept of hemostatic resuscitation has changed the practical approach to trauma patients. Based initially on a retrospective review of military resuscitation practices, researchers have come to understand that a balanced hemostatic resuscitation regimen targeting repletion of lost whole blood through replacement with a balanced (of at least 1:2) ratio of plasma and platelets, in addition to red cells, decreases mortality and morbidity [4].

## Implicating trauma, not resuscitation

A decade ago, the notion was widespread that patients experienced coagulopathy solely as an unfortunate iatrogenic effect of well-meaning resuscitative practices. The pioneering shock research of the 1970s led to an emphasis on improving tissue perfusion, in the form of blood flow and oxygen-carrying capacity, as the primary goal of resuscitation. This belief, combined with the separation of blood components by blood banks in the 1970s (at first to improve resource allocation and then to limit transmission of blood-borne infections such as hepatitis C and HIV), resulted in decades of treatment in which trauma patients received large volumes of crystalloid and red blood cells at the expense of perturbations in coagulation and inflammation. When iatrogenic complications resulted, damage control surgery aimed at providing countermeasures, but the root cause of coagulopathy remained eclipsed by the primary emphasis on perfusion.

Subsequent research has established the understanding of ATC as an endogenous perturbation of coagulation that occurs as a result of injury and shock nearly immediately after trauma. Initial characterization and basic investigation have revealed that a primary driver of this coagulopathy is activation of the protein C system leading to the proteolytic cleavage of factors Va and VIIa, as well as the derepression of fibrinolysis, resulting in a coagulopathic state characterized by bleeding, morbidity, and mortality [3,5,6]. Further work on sterile inflammation led to the important realization that perturbations after trauma do not simply represent impaired or ineffective clot formation. Rather, we now understand that ATC involves a perturbation of coagulation, inflammation, and innate immunity, including the complex interactions among these processes. This biological conception can explain why, while exsanguination from massive hemorrhage is uncommon in the setting of modern prehospital treatment, fast transport to definitive care, and advanced surgical capabilities, some patients still die from bleeding. Death from ongoing blood loss among such patients who initially survive to physical hemorrhage control and “definitive” care can be understood as the consequence of having exceeded a biologic or physiologic threshold of irreversible dysfunction in inflammation or coagulation. Understanding how to mitigate this dysregulated biology of processes involving the lining of damaged blood vessels (endotheliopathy) is the essential future work for this field.

## Targeting endotheliopathy

Understanding the endotheliopathy of trauma can facilitate targeting more effectively the coagulation and inflammation disturbances of severely injured patients. The important progress in hemostatic resuscitation has laid the groundwork, with significant mortality and morbidity reductions. Nonetheless, attempts at definitive trials have been negative and have failed to show benefit in the heterogeneous population of patients with severe traumatic injury [7]. Evidently, a “one-size-fits-all” approach to hemostatic resuscitation under-resuscitates some and over-resuscitates others. In the era of the advent of personalized medicine, it is now essential to understand each patient as possessing individual dynamic physiologic states that should be individually targeted with specific therapies [8]. Recent work suggests that big data approaches and dynamic modeling can identify and track physiologic states and predict clinical trajectories, raising the exciting possibility of providing decision support and driving individualized dynamic treatment [9–12].

Future work in this evolving area should center around 2 areas. First is solidifying the basic understanding of postinjury biology and physiology. Multiple groups in North America and Europe are examining crucial aspects of this work, including the protein C system, endothelial dysfunction, platelet function, damage-associated molecular pattern (DAMP)-related coagulation, and activation of fibrinolysis, among other topics. Only through comprehensive characterization from patient sampling and meticulous collection of physiologic and outcome data combined with rigorous animal and molecular science can we fully understand the biology underlying how trauma patients respond to environment, injury, and resuscitation and how to translate this understanding into more insightful care that truly saves lives.

## Abbreviations

ATC: acute traumatic coagulopathy
DAMP: damage-associated molecular pattern
TIC: trauma-induced coagulopathy
